# A novel way to remove a broken intramedullary nail

**DOI:** 10.1308/003588412X13373405387096h

**Published:** 2012-05

**Authors:** G Smith, A Khan, A Marsh

**Affiliations:** Dudley Group NHS Foundation Trust,UK

## BACKGROUND

Removal of broken intramedullary nails can prove challenging and several techniques have been suggested to achieve retrieval.^1^ The increasing use of retrograde nails for calcaneotalotibial arthrodesis has a particular problem: the cannulation is often too narrow to allow passage of hooks or other commonly used extraction devices.^2^ We propose a method using equipment that is readily available to aid removal of a deeply placed proximal fragment.

## TECHNIQUE

**Figure 1:**
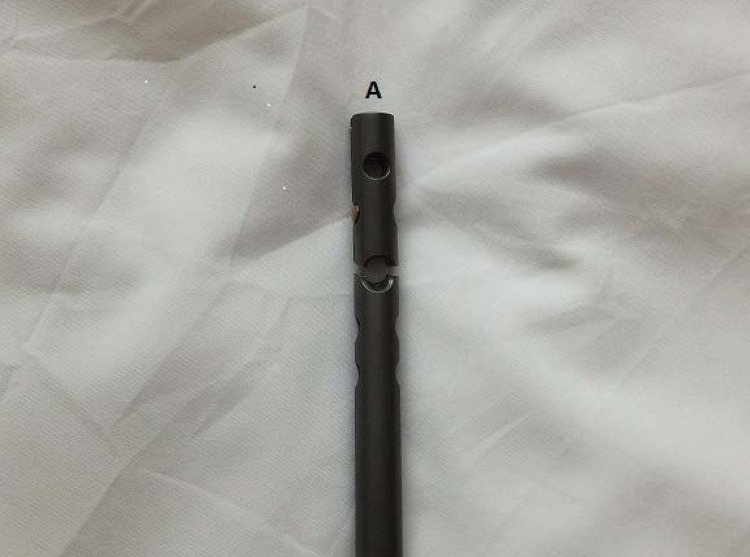
The fragment closest to the insertion that requires removal first

**Figure 2:**
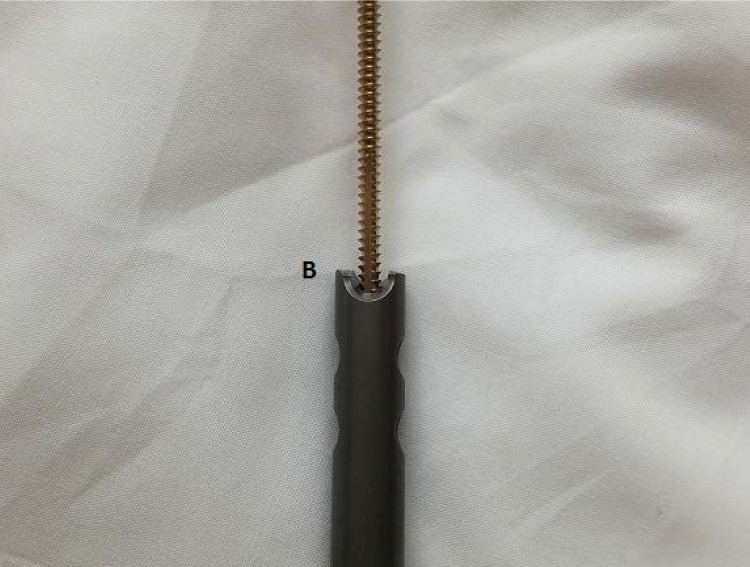
The 3.5mm tap inserted into the cannulation of the nail

**Figure 3:**
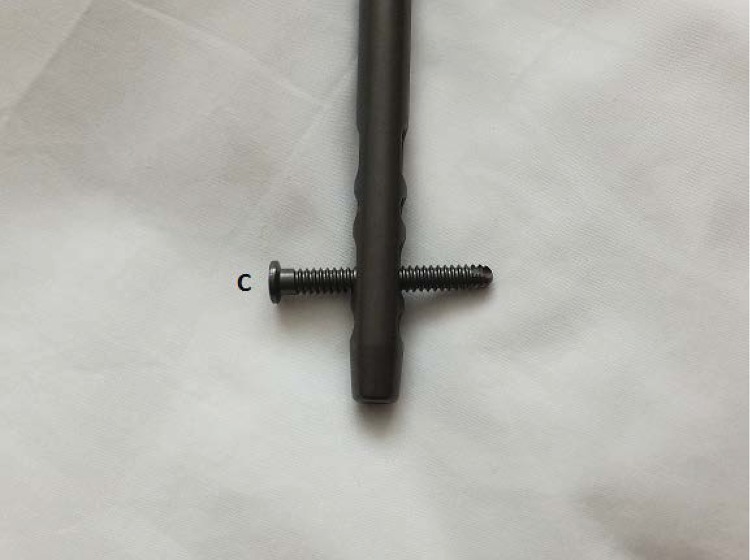
The transverse locking bolt that needs to be removed before the nail can be extracted

First, remove the fragment closest to the insertion incision using standard extraction equipment (Fig 1). To remove the more distant broken part of the nail, a small fragment 3.5mm tap is inserted by hand into the cannulation of the nail (Fig 2) before removing any transverse locking bolts (to prevent the fragment rotating). After the tap has ‘grabbed’ the fragment, the locking bolts can be removed (Fig 3) and the fragment withdrawn through the bone tunnel. As the tap is made of steel, it will engage inside the nail (usually made of titanium alloy), allowing it to be removed.

## DISCUSSION

This technique allows minimally invasive extraction of a broken fragment without having to window the bone in which it is located. Nails with a larger diameter cannulation can be extracted in a similar way with a larger diameter tap.
